# Brain proteome changes after intracerebral hemorrhage in aged male and female mice

**DOI:** 10.1016/j.nbd.2025.106936

**Published:** 2025-05-02

**Authors:** Sivaraman Kuppuswamy, Noah J. Watson, William Luke Ledford, Blake A. Pavri, Wenbo Zhi, Mary Gbadebo, Frederick Bonsack, Hongyan Xu, Sangeetha Sukumari-Ramesh

**Affiliations:** Department of Pharmacology and Toxicology, Medical College of Georgia, Augusta University, 1120, 15th Street, CB3515, Augusta, GA 30912, United States of America

**Keywords:** Intracerebral hemorrhage, Aging, Hematoma, Mass spectrometry, Bioinformatics

## Abstract

Aging is an independent predictor of adverse outcomes after intracerebral hemorrhage (ICH), a stroke subtype with no effective treatment. Despite the expected increase in the incidence of ICH due to population aging and the widespread use of anticoagulants, preclinical studies with aged animal subjects are lacking, and the pathophysiology of ICH in aged subjects has yet to be defined. Herein, we attempt to characterize the brain proteomic changes after ICH using an unbiased label- free quantitative proteomics approach and bioinformatics. To this end, aged male and female mice (18–24 months old) were subjected to sham/ICH. Mice were euthanized on day 3 post-surgery, and ipsilateral brain tissue was collected and subjected to LC-MS/MS analysis. Considering sex as a biological variable, the data derived from males and females were separately analyzed. The proteomics analysis revealed 133 differentially expressed proteins (DEPs) between the sham and ICH groups in male subjects. Among the DEPs, 98 proteins were downregulated, and 35 proteins were upregulated after ICH, compared to sham. In aged female mice, 315 DEPs were identified, of which 221 proteins were downregulated, and 94 proteins were upregulated after ICH compared to sham. The mass spectrometry data was validated using immunohistochemistry or western blot analysis, and the bioinformatics analysis revealed a comprehensive understanding of the signaling pathways associated with ICH. Some DEPs in both aged male and female mice that could play roles in ICH pathology were 14–3-3 proteins and S100-A9. The study also revealed that mitochondrial dysfunction could be a critical regulator of ICH-induced acute brain damage. Overall, the generated proteomics data could help develop hypothesis-driven functional analysis and delineate the complex pathobiology of ICH.

## 1. Introduction

Intracerebral hemorrhage (ICH) is a stroke subtype and a major public health concern with high mortality and morbidity rates (Feigin et al., 2021; O’Carroll et al., 2021). Per the Global Burden of Disease Study, 3.4 million suffered intracerebral hemorrhage globally in 2019, resulting in 2.8 million deaths (Feigin et al., 2021). Despite high death rates, the ICH survivors often live with disabilities, causing a substantial socio-economic burden. The major risk factors of ICH are advanced age, hypertension, and cerebral amyloid angiopathy (Feldmann et al., 2005; Morotti and Goldstein, 2016). Among these, advanced age is also an independent predictor of adverse outcomes in patients with ICH (Hemphill 3rd et al., 2001), and older patients have a higher 30-day mortality rate (González-Pérez et al., 2013). Also, there was a remarkable increase in age-related ICH cases per 100,000 individuals, from 5.9 in the 35–54-year-old group to 176.3 in the 75–94-year-old group (Jolink et al., 2015). Importantly, besides age-associated changes in health conditions, the aged population often presents with comorbidities such as hypertension (Ariesen et al., 2003), contributing to the risk and pathophysiology of ICH.

Despite the emerging advances in preclinical studies, an effective treatment for ICH has yet to be established, owing partly to the complexity of the disorder. ICH pathology comprises primary and secondary brain insults. The extravasation of blood and the mass effect of hematoma contribute to primary brain damage, whereas the cellular and molecular responses to primary injury culminate in secondary brain damage. The secondary brain injury mechanisms include but are not limited to neuroinflammation, oxidative stress, and blood-induced neurotoxicity. Though preclinical ICH models could help partly delineate the disease pathology, studies were conducted largely on young and male animal subjects. Therefore, preclinical studies with aged animal subjects are imperative to better understand the underlying pathophysiology of ICH-induced brain damage. There is no sex difference in the incidence of ICH per clinical studies (Gokhale et al., 2015); however, a few preclinical studies to date employed female subjects. Herein, using a well-established preclinical model of ICH, we attempt to characterize the brain proteomic changes after ICH in old animal subjects using an unbiased label-free quantitative proteomic (LC-MS/MS: Liquid Chromatography Tandem-Mass Spectrometry) and bioinformatics approaches. The study design incorporated male and female subjects, and the data derived from males and females were analyzed separately. Overall, the mass-spectrometry-based proteomics approach provides compelling opportunities to characterize the CNS complexity post-injury and identify potential therapeutic targets for functional characterization.

## 2. Materials and methods

### 2.1. Mice

C57BL/6 male and female aged mice (18–24 months old, National Institute of Aging, USA) were housed under standard, pathogen-free conditions and light-dark cycles (12-h light and 12-h dark), with free access to food and water. All animal procedures were reviewed and approved by the Institutional Animal Care and Use Committee.

### 2.2. Induction of ICH

ICH was induced in aged mice, as previously reported (Bonsack et al., 2016; Ramesh et al., 2012; Sukumari-Ramesh et al., 2012). Briefly, the mice (C57BL/6 male and female) were anesthetized with isoflurane (3 % induction and 2 % maintenance), a burr hole (0.5 mm) was made 2.2 mm lateral to the bregma, and the mice were positioned on a stereotaxic frame. Throughout the surgical procedure, aseptic techniques were followed, and the body temperature was maintained (37 ± 0.5 °C). Using stereotactic guidance, a syringe (Hamilton, 26-G) containing bacterial collagenase (0.04 U in 0.5 μL Phosphate Buffered Saline, pH 7.4; PBS) was inserted 3.5 mm deep into the left striatum, and collagenase was injected to induce ICH. The needle was removed gently, and the incision was closed. The same procedure was followed for experimental control/sham mice, on which instead of collagenase, PBS (0.5 μL) was injected into the striatum.

### 2.3. Sample preparation and LC-MS/MS analysis

**Fig. 1.**
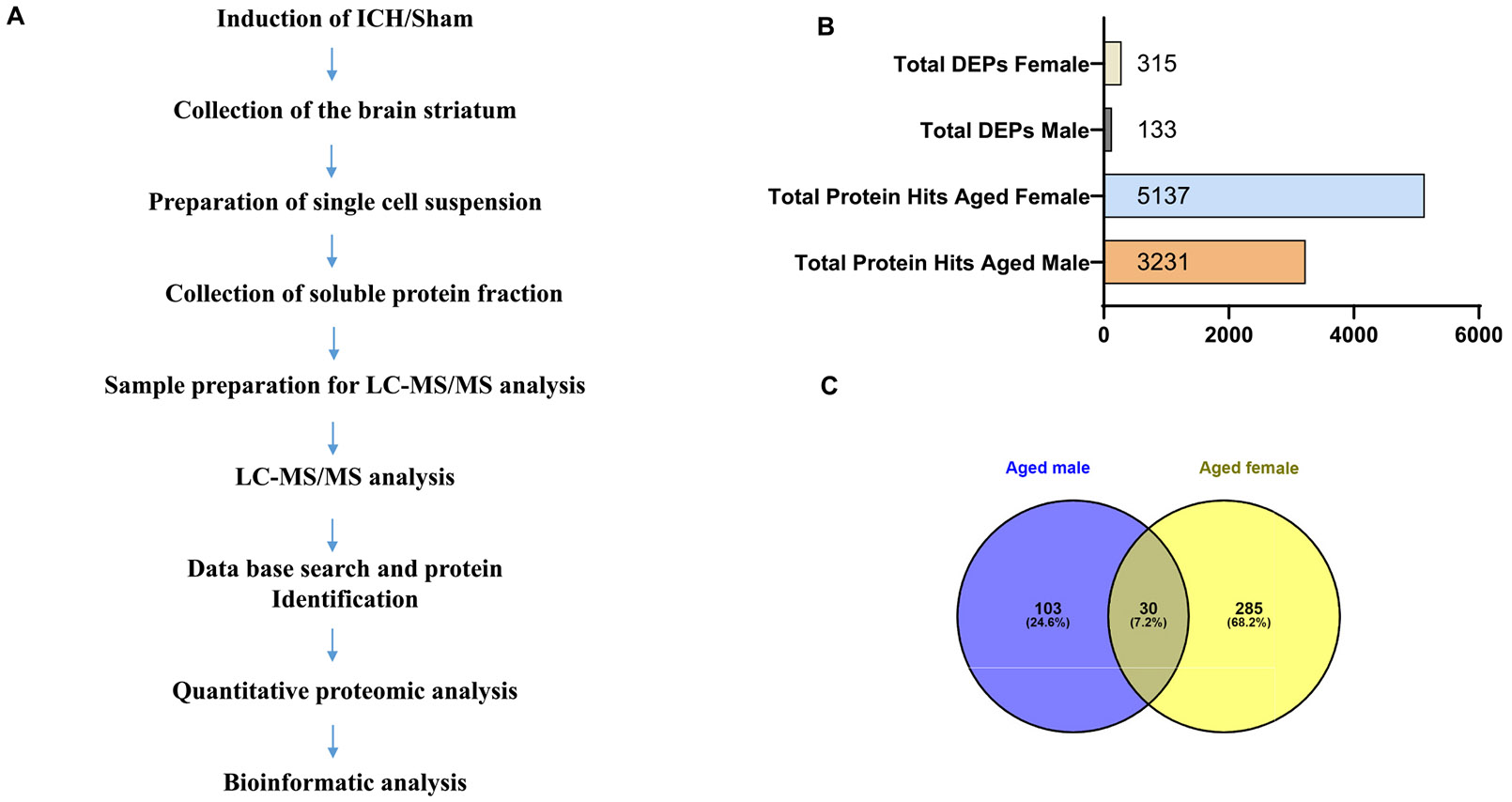
An overview of protein identification by mass spectrometry. Aged male and female mice were subjected to ICH or sham, and at day 3 post-surgery, ipsilateral brain regions were collected and subjected to mass spectrometry. (A) An illustration of the workflow and (B) depicts the total number of proteins identified and differentially expressed in the aged male and female subjects. (C) Venn diagram illustrating the number of common and unique DEPs in aged male and female subjects (*n* = 4–5/group).

As previously reported (Dasari et al., 2020) and depicted in Fig. 1A, the brain samples were collected after ICH or sham and processed for mass spectrometry (LC-MS/MS analysis). Briefly, mice were anesthetized with isoflurane and transcardially perfused with ice-cold PBS, and the striatum containing both the hematoma and perihematomal brain tissues was collected from ICH mice. The respective brain tissue from sham animals served as the experimental control. This was followed by collecting single-cell suspension in ice-cold PBS containing protease and phosphatase inhibitors. For this, the brain tissue was placed on a 100 μm cell strainer and gently passed through using a syringe plunger. The single-cell suspension in ice-cold PBS containing protease and phosphatase inhibitors was then centrifuged at 1000 rpm for 5 min, and the cell pellet was collected, which was subsequently sonicated and then centrifuged at 14,000 rpm for 5 min to collect the soluble fraction. After measuring the protein concentration, 100 μg of total protein per sample was precipitated by adding 8 times the volume of cold acetone and 1 time the volume of 100 % trichloroacetic acid (TCA). The protein precipitates were then washed with cold acetone, air dried, and reconstituted in 40 μl of 8 M urea in 50 mM Tris-HCl (pH 8). This was followed by treatment with 10 mM DTT and 55 mM iodoacetamide. Then, 360 μl of 50 mM ammonium bicarbonate buffer was added to reduce the urea concentration. For the LC-MS/MS analysis, the protein digestion was carried out using a 1:20 ratio (*w*/w) of Trypsin (Pierce, Rockford, IL) at 37 ^◦^C overnight. Trypsin-digested samples were then cleaned using a C18 micro-spin plate (Harvard Apparatus) before LC-MS/MS analysis.

LC-MS/MS analysis was performed as previously reported (Sharma et al., 2018) at the proteomics core facility at Augusta University, Briefly, the peptide samples were washed using a Pepmap100 C18 trap (5 μm, 0.3 × 5 mm) at 20 μL/min with 2 % acetonitrile in water (with 0.1 % formic acid) for 10 min and separated using a Pepman100 RSLC C18 column (2.0 μm, 75-μm × 150-mm) with acetonitrile (2 to 40 %) and formic acid (0.1 %) at a flow rate of 300 nL/min at a column temperature of 40 °C. Eluted peptides were injected into an Orbitrap Fusion MS via nano-electrospray ionization using temperature and voltage, 300 ° C and 2000 V, respectively. The peptides were analyzed by data-dependent acquisition (positive mode) using an Orbitrap MS analyzer for precursor scan (120,000 FWHM from 300 to 1500 *m/z*) and an ion-trap MS analyzer for MS/MS scans in the speed mode with exclusion settings as reported previously (Sharma et al., 2018). Higher-energy collisional dissociation (HCD) was used as a fragmentation method with a normalized collision energy of 32 %.

The raw MS and MS/MS spectra were processed using the Proteome Discoverer software by Thermo Scientific (v1.4) and searched against the Uniport mouse database using the SequestHT search algorithm (precursor ion mass tolerance: 10 ppm, product ion mass tolerance: 0.6 Da, static Carbamidomethylation of +57.021 Da of cysteine and dynamic oxidation for methionine (+15.995 Da). The Percolator PSM validator algorithm was used to validate the peptide spectrum match and estimate the false discovery rate to be *<*1 % (q-Value). Proteins unable to be identified and distinguished based on the database search results alone were grouped to satisfy the principles of parsimony. A report containing the protein identities and spectrum counts (number of peptide spectrum matches (PSM)) for each protein was generated, and spectral counting-based quantitative analysis was performed.

For quantitative analysis of the LC-MS/MS data, each identified protein’s PSM count, a semi-quantitative measure of the protein, was normalized against the total PSM counts of all identified proteins in that sample, and the average PSM count of replicates was subjected to statistical analysis. Power analysis and sample size estimation were performed using the statistical software R v4.1.2. Based on our prior mass spectrometry-based quantitative proteomic study (Dasari et al., 2020), the mean effect size (4.731) was calculated, and to detect the mean effect size, the required sample size is 3, which would yield a power of 80 % at a 0.05 significance level.

### 2.4. Western blotting

As reported earlier (Dasari et al., 2020), the brain tissue was collected for western blotting in RIPA buffer in the presence of protease and phosphatase inhibitors. After sonication, the samples were centrifuged, and the fifty micrograms supernatant was subjected to a gradient SDS-PAGE gel (4–20 %) electrophoresis and transferred onto a PVDF membrane. After blocking the membrane, incubations were carried out with the respective primary antibody (14–3-3 (pan) antibody, catalog number: 8312, Cell Signaling Technology, USA, or 14–3-3 gamma antibody, catalog number: ab155050, Abcam, USA, or Catalase antibody, Catalog number: ab52477, Abcam, USA, or Glutathione Peroxidase 1 antibody, Catalog number ab22604, Abcam, USA, or beta-actin antibody, catalog number: A2228, Sigma, USA) and subsequently, with the respective fluorescent-tagged secondary antibody [Alexa fluor 750 goat anti-rabbit IgG; catalog: A21039; Invitrogen, USA or Alexa fluor 750 goat anti-mouse IgG; catalog number: A21037; Invitrogen, USA). The blots were imaged using iBright 1500 (Thermofisher, USA), and for quantification, the acquired images were analyzed using ImageJ software with beta-actin as a loading control.

### 2.5. Immunohistochemistry

The mice were anesthetized and transcardially perfused with ice-cold PBS. The collected brains were fixed overnight, cryoprotected, and embedded into OCT. The brains were then snap-frozen and sectioned. As previously reported (Bonsack et al., 2016), brain sections (20 μm) were permeabilized and blocked with donkey serum, which was followed by incubation with respective primary antibody (S100A9 antibody; catalog number: ab242945; Abcam, USA, or glial fibrillary acidic protein (GFAP) antibody; catalog number: ab53554; Abcam, USA) at 4 °C overnight and secondary antibody (Alexa Fluor 488 donkey anti-rabbit IgG; catalog number: A-21206; Invitrogen, USA, or Alexa Fluor 488 donkey anti-goat IgG; catalog number: A-11055; Invitrogen, USA) at room temperature for 1 h. After washing and applying a mounting medium containing DAPI (DAPI Fluoromount-G; SouthernBiotech, USA), the sections were cover-slipped and imaged using an inverted confocal laser microscope (Zeiss 780).

### 2.6. Bioinformatics analysis

PANTHER (Protein Analysis Through Evolutionary Relationships) classification system and Qiagen IPA (Ingenuity Pathway Analysis) were used for bioinformatics analysis.

### 2.7. Statistical analysis

The difference between the two groups was assessed using the student’s *t*-test. A *p <* 0.05 was statistically significant.

## 3. Results

### 3.1. Identification of differentially expressed proteins (DEPs) after ICH

**Fig. 2.**
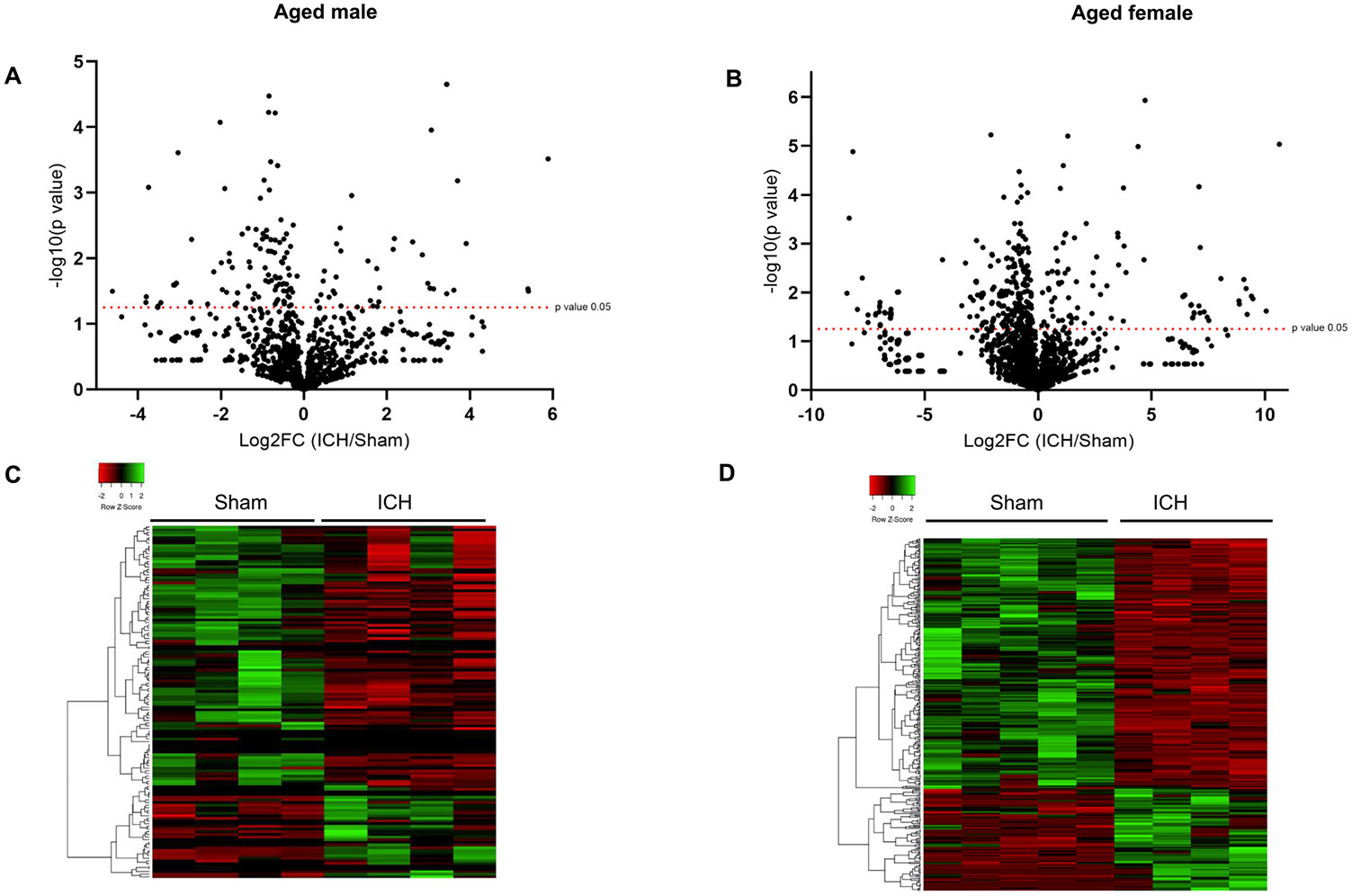
Volcano plot and heatmap demonstrating the deregulated expression of proteins after ICH. The volcano plots (A) and (B) denote the distribution of DEPs in aged male and female subjects, respectively, with the horizontal dotted line representing the *p*-value of 0.05, and all proteins above that line are significantly deregulated after ICH compared to sham. The heat maps (generated using http://www.heatmapper.ca), (C), and (D) illustrate the expression pattern of DEPs in aged male and female subjects, respectively (*n* = 4–5/group).

ICH was induced in both aged male and female mice (C57BL/6) by stereotactically injecting collagenase into the mouse brain striatum, per the methods section. The brain tissue was collected for mass spectrometry analysis at day 3 post-injury, an acute time point that exhibited profound glial activation (Bonsack et al., 2016; Ramesh et al., 2012; Sukumari-Ramesh et al., 2012), a key regulator of secondary brain damage. For a better signal-to-noise ratio, a single-cell suspension of the ipsilateral brain region was prepared as detailed in the methods section and subjected to LC-MS/MS analysis. Age- and sex-matched mice were subjected to sham surgery and the ipsilateral brain region from the sham animals served as the experimental control. The data derived from male and female subjects were analyzed separately, considering sex as a biological variable. The proteomics analysis of the brain tissue derived from aged male mice post-surgery revealed the expression of 3231 proteins between the sham and ICH groups. Of those, as per the quantitative proteomics analysis, there were 133 differentially expressed proteins (DEPs) between sham and ICH groups (*p* value *<*0.05), which included 98 significantly downregulated proteins and 35 significantly upregulated proteins in the ICH group compared to sham (Fig. 1B). The proteomics data from the female mice brain samples revealed 5137 proteins between the sham and ICH groups (Fig. 1B). The quantitative analysis revealed 315 DEPs between the two groups (*p* value *<*0.05), of which 94 proteins were upregulated, and 221 proteins were down-regulated post-ICH compared to sham (Fig. 1B). The augmented levels of brain hemoglobin subunits (alpha and beta-1) in the ICH groups confirmed the induction of ICH in both male and female subjects. The comparative analysis of DEPs derived from male and female subjects using the Venn diagram demonstrates the number of common and unique DEPs between the male and female subjects (Fig. 1C). Fig. 2 illustrates the distribution and expression pattern of DEPs as per the volcano plot and heat map, respectively. The complete list of DEPs from male and female subjects is provided in Supplementary Tables 1 and 2, respectively. Also, the common DEPs from male and female subjects are provided in Supplementary Table 3.

### 3.2. Validation of proteomics data

**Fig. 3.**
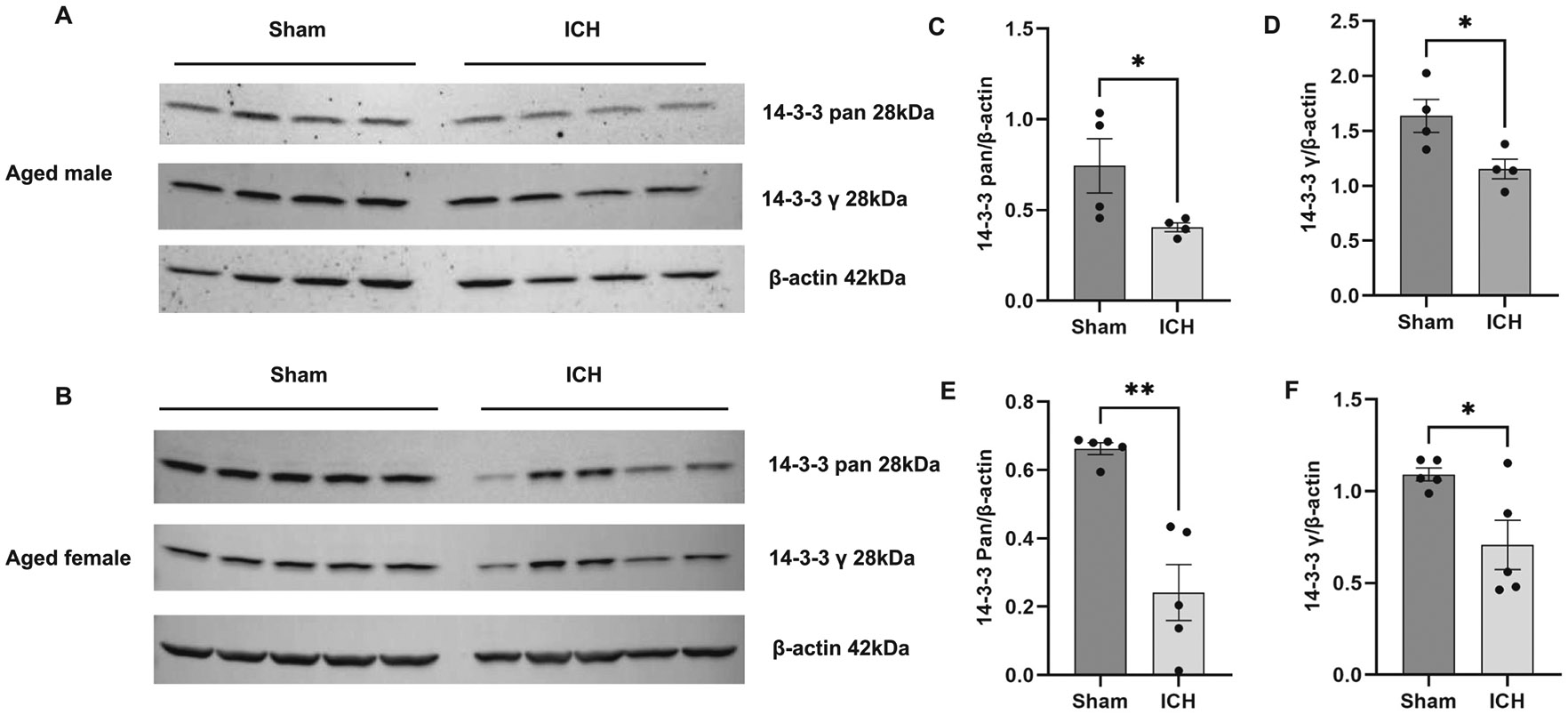
Validation of quantitative mass spectrometry analysis. The western blotting analysis demonstrates the brain expression level of protein candidates, 14–3-3 (pan) and 14–3-3 gamma isoform, in aged male subjects (A) and aged female subjects (B) after ICH or sham. (C & D) and (E &F) illustrate the densitometry analysis of the western blotting data normalized against beta-actin from aged male and female subjects, respectively (*n* = 4–5/group).

**Fig. 4.**
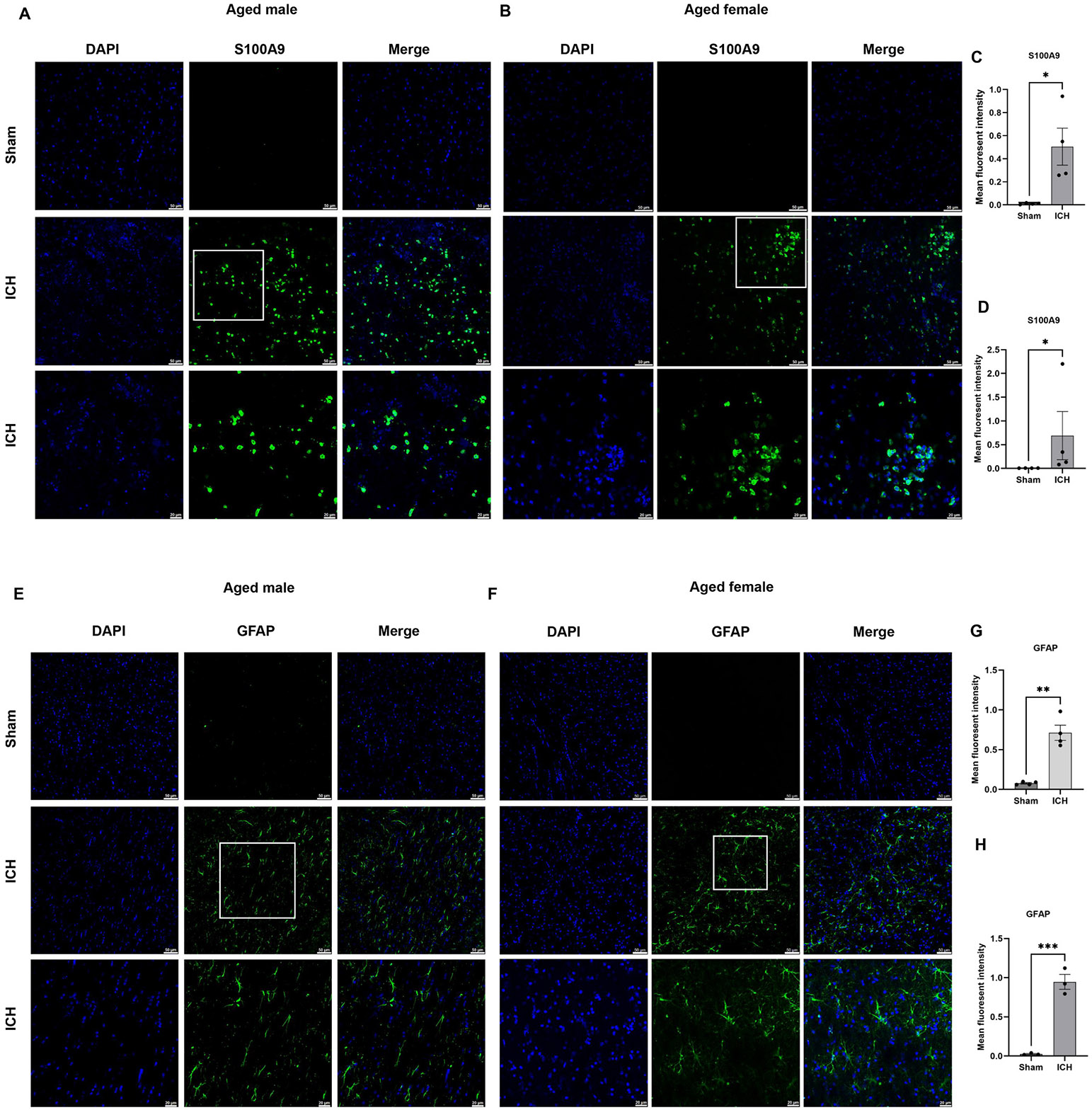
Validation of quantitative mass spectrometry analysis. The immunohistochemistry analysis illustrates the expression of S100A9 in the brain striatum in aged male subjects (A) and aged female subjects (B) after ICH or sham. (C & D) demonstrate the mean fluorescence intensity of S100A9 expression in aged male and aged female subjects, respectively, analyzed with ImageJ software (NIH USA). (E&F) demonstrates the ICH-induced expression of GFAP in the brain striatum, and (G&H) denotes the mean fluorescence intensity of GFAP expression in aged male and female subjects, respectively, analyzed with ImageJ software (NIH USA) (n = 4/group).

**Fig. 5.**
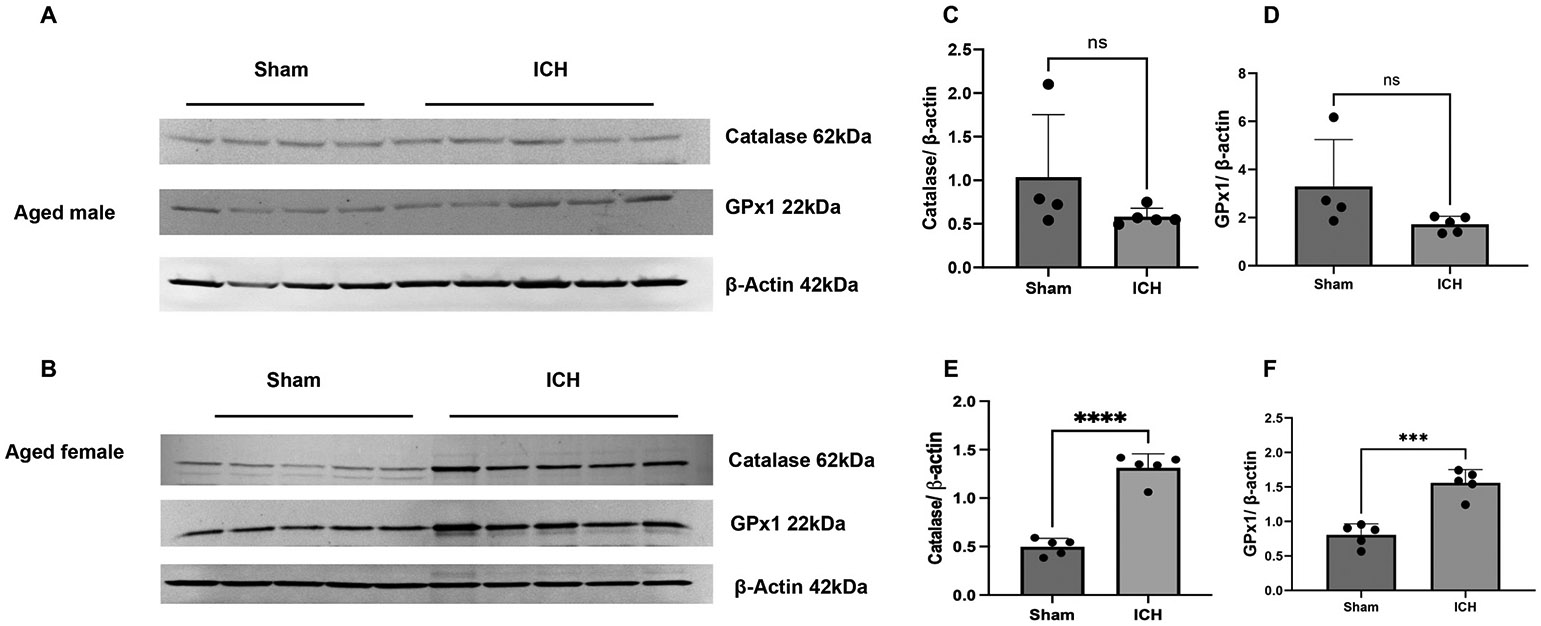
Validation of quantitative mass spectrometry analysis. The western blotting analysis demonstrates the brain expression level of catalase and glutathione peroxidase 1 (GPx1) in aged male subjects (A) and aged female subjects (B) after ICH or sham. (C & D) and (E &F) illustrate the densitometry analysis of the western blotting data normalized against beta-actin from aged male and female subjects, respectively (n = 4–5/group).

To validate the proteomics data, six candidate proteins from the male experimental group and six from the female were selected, and western blot analysis or immunohistochemistry was performed. The selection of the candidate proteins for validation was based on their potential to be considered for functional studies (14–3-3 and S100A9) and to reveal additional novel information derived from quantitative proteomics, such that there could be sex-based differences in antioxidant response after ICH. 14–3-3 proteins comprise a number of isoforms and can serve as hub proteins regulating a wide range of biological processes and, hence, could play roles in ICH pathology. As per the quantitative proteomics analysis, several 14–3-3 isoforms were found to be down-regulated in both aged male and female mice after ICH, so first, we used a pan antibody against 14–3-3, which recognizes all isoforms of mammalian 14–3-3 proteins (β/α, γ, ε, η, ζ/δ, θ/τ and σ). In addition, using an isoform-specific antibody, we also evaluated the expression of one of the isoforms, 14–3-3 gamma (γ), which could play a role in brain damage (Dong et al., 2010; Nelson and Alkon, 2007). Consistent with the proteomics analysis, the western blotting data demonstrated the downregulation of 14–3-3 and 14–3-3 gamma (Fig. 3). Also, in line with quantitative proteomics analysis, the expression of S100A9, an inflammatory mediator and novel candidate to be tested for its role after ICH and GFAP (Glial fibrillary acidic protein), an established marker of injury-induced astrogliosis, were upregulated in aged male and female ICH mice compared to sham as evidenced by immunohistochemistry (Fig. 4). Moreover, consistent with quantitative proteomics analysis, the expression of antioxidant proteins, catalase, and glutathione peroxidase 1 was significantly upregulated in female-aged mice after ICH (Fig. 5). However, there was no change in catalase and glutathione peroxidase expression in aged male subjects after ICH compared to sham (Fig. 5), further validating the quantitative proteomic analysis.

### 3.3. Functional characterization of DEPs

**Fig. 6.**
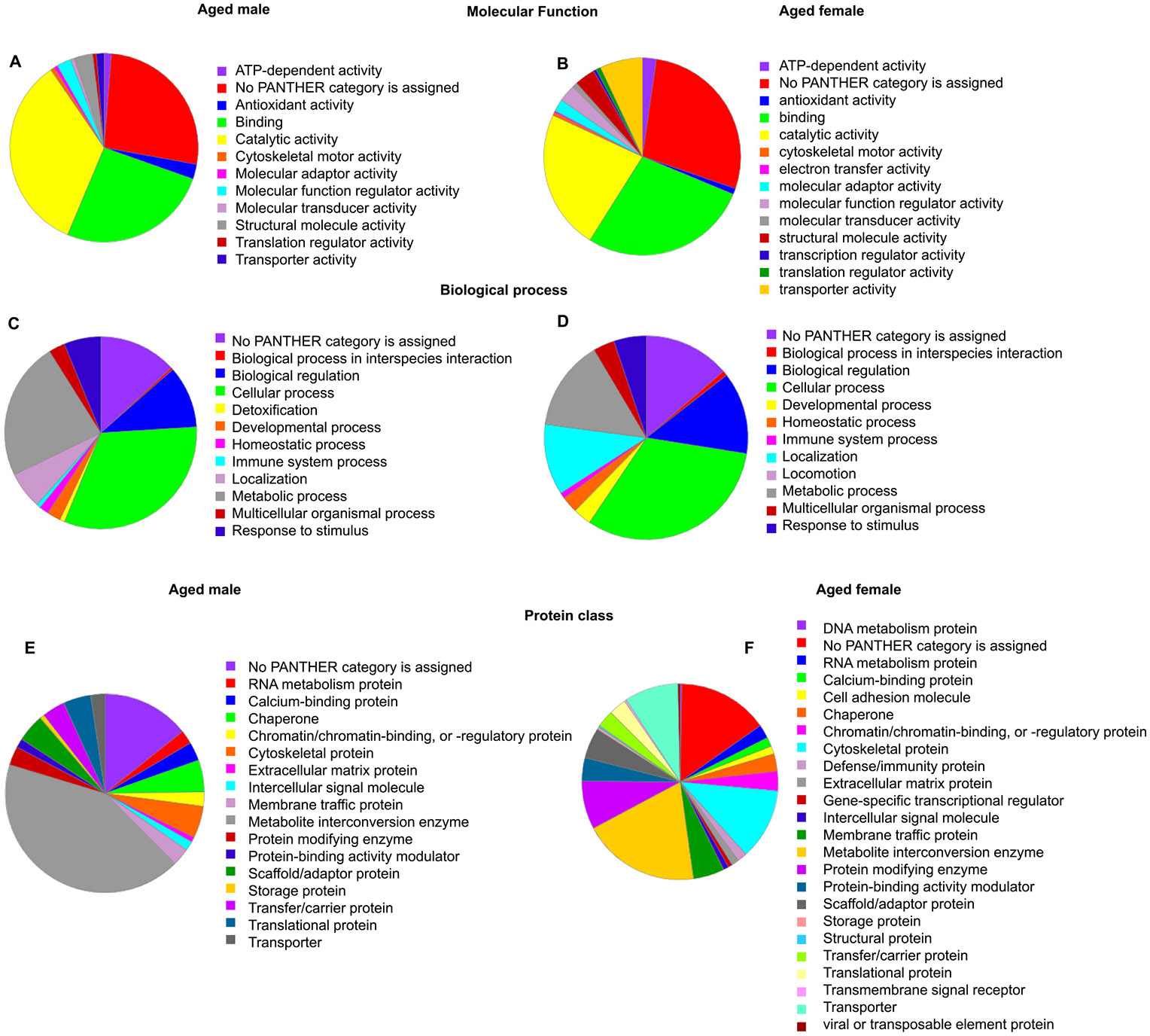
The bioinformatics analysis of DEPs. The gene ontology analysis by PANTHER illustrates molecular function (A&B), biological process (C&D), and protein class (E&F) associated with the DEPs in aged male and female subjects, respectively.

To gain insights into the functional roles of the differentially expressed proteins, the Uniport accession numbers of DEPs were evaluated using a bioinformatics application, PANTHER (Mi et al., 2010), against the mouse protein ontology database. Based on the analysis, the predominant molecular functions that the DEPs from the male subjects carried were catalytic activity (40.6 %) and binding (30.8 %) (Fig. 6). The major biological processes associated with the DEPs were cellular process, metabolic process, biological regulation, localization, and response to stimulus. The prominent protein classes the DEP belonged to were metabolite interconversion enzyme, chaperone, cytoskeletal protein, scaffold/adaptor protein, and translational protein (Fig. 6).

The predominant molecular functions that the DEPs from the female subjects carried were catalytic activity (28.3 %), binding (34.1 %), and transporter activity (8.6 %) (Fig. 6). The top biological processes associated with the DEP were cellular process, metabolic process, and biological regulation. The prominent protein classes the DEPs belonged to were metabolite interconversion enzyme, cytoskeletal protein, transporter, protein modifying enzyme, membrane traffic protein, and scaffold/adaptor protein (Fig. 6).

### 3.4. Identification of signaling pathways associated with the DEPs

**Fig. 7.**
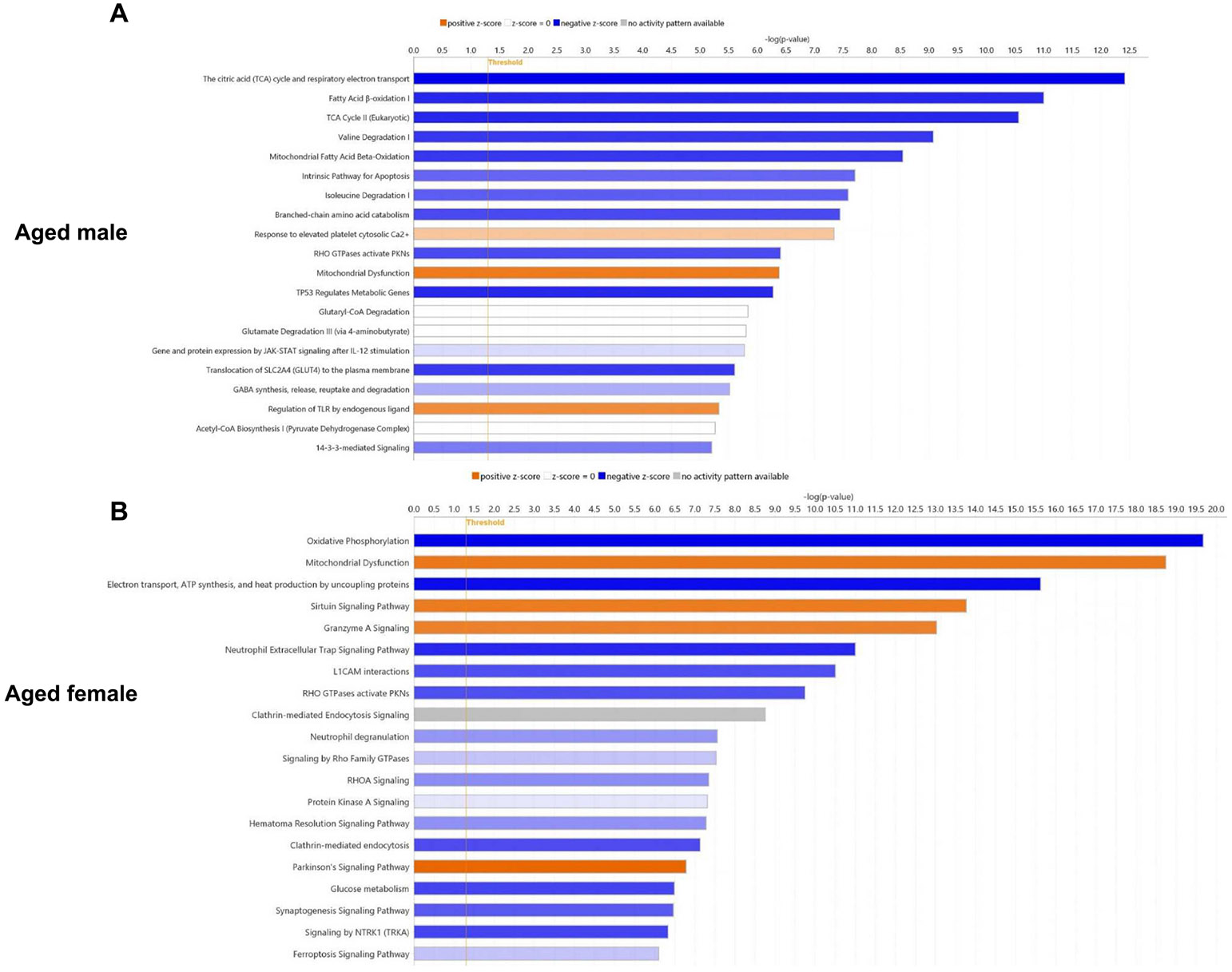
Top signaling pathways identified using IPA analysis. (A) and (B) denotes the top 20 signaling pathways associated with the DEPs from aged male and female subjects, respectively.

To identify the signaling pathways associated with DEPs, Ingenuity Pathway Analysis (IPA, Qiagen), a powerful tool for the analysis and interpretation of omics data, was used. Per the analysis, some of the prominent pathways associated with the DEPs from the male subjects are mitochondrial dysfunction, the intrinsic pathway for apoptosis, response to elevated platelet cytosolic Ca2+, regulation of TLR by endogenous ligand, 14–3-3 mediated signaling, and Neuroinflammation Signaling Pathway. The top 20 canonical pathways identified are listed in Fig. 7 and the DEPs associated with key pathways are listed in Table 1. Some of the key DEPs associated with the aforementioned pathways are Aconitate hydratase, ATP synthase subunit g, Superoxide dismutase [Cu—Zn] (SOD1), Superoxide dismutase [Mn] (SOD2), 14–3-3 protein beta/alpha, 14–3-3 protein gamma, 14–3-3 protein eta, 14–3-3 protein theta, and Protein S100-A9. The complete list of pathways identified is provided in Supplementary Table 4.

Some of the prominent pathways associated with the DEPs from the female subjects are mitochondrial dysfunction, sirtuin signaling pathway, neutrophil extracellular trap signaling pathway, clathrin mediated endocytosis, glucose metabolism, ferroptosis signaling pathway, and 14–3-3 mediated signaling. The top 20 canonical pathways identified are listed in Fig. 7 and the DEPs associated with key pathways are listed in Table 2. Some of the key DEPs associated with the aforementioned pathways are Glutathione peroxidase 1, Voltage-dependent anion-selective channel protein 1, Toll-like receptor 7, Hexokinase-1, Serotransferrin, 14–3-3 protein beta/alpha, 14–3-3 protein epsilon, 14–3-3 protein gamma, 14–3-3 protein theta, and 14–3-3 protein zeta/delta. The complete list of pathways identified is provided in Supplementary Table 5. Despite the difference in ranking per the IPA analysis, the above-mentioned pathways identified in the female subjects were also identified in males and vice versa.

## 4. Discussion

ICH is a deadly stroke subtype with survivors often exhibiting lifelong disabilities (Bhalla et al., 2013; Ferro, 2006). Given the increased prevalence of ICH in the elderly population coupled with the lack of effective treatment options, a comprehensive understanding of the proteomic changes in the aged brain after ICH is imperative. In the present study, we employed a label-free quantitative proteomics method. Based on the data extraction method after the label-free proteomics approach, the data quantification can be performed using the spectral counting method (PSM method) or area under the curve (AUC) method. The present study used the spectral counting method, which is ideal and widely used for discovery experiments and assesses the number of identified MS/MS spectra matches (peptide spectrum match or PSM) of a protein. The spectral counting method is more reproducible (Zybailov et al., 2005), easy to implement, and the number of spectra directly correlates to relative protein abundance (r2 = 0.9997) (Liu et al., 2004). In contrast, the AUC method measures the area of the precursor ions’ chromatographic peaks and requires delicate and automated algorithms and software for data analysis (Zhu et al., 2010). Moreover, there could be run-to-run variations, and any change in retention time and *m*/*z* can cause unaligned peaks, complicating the data analysis with the AUC method (Zhu et al., 2010). Employing an unbiased proteomics approach, the present study identified several differentially expressed proteins in the ipsilateral brain region of aged male and female mice post-ICH compared to control. Also, the bioinformatics analysis revealed various pathological processes and signaling pathways associated with the differentially expressed proteins that could play roles after ICH.

Extravasated blood components and hemoglobin metabolites accumulate in the brain after ICH and a multitude of intra and extra-cellular mechanisms contribute to ICH-indued brain damage (Madangarli et al., 2019; Watson et al., 2022). As per the proteomics and bioinformatics analysis mitochondrial dysfunction is one of the prominent mechanisms associated with ICH in both aged male and female mice, and the expression of several mitochondrial function-associated proteins were found to be altered after ICH. As per previous studies, oxygen consumption is found to be reduced in the perihematomal region of ICH patients from 6 h to 72 h post-ICH (Sook Kim-Han et al., 2006). Notably, mechanisms other than ischemia could be contributing to mitochondrial dysfunction since cerebrovascular autoregulation was intact in the perihematomal region in ICH patients (Sook Kim-Han et al., 2006). Mitochondria is a highly dynamic organelle that undergoes fission and fusion. The function of mitochondria depends largely on the dynamic balance between fission and fusion, which is often found to be disrupted in various pathological conditions (Burté et al., 2015; El-Hattab et al., 2017). Besides regulating neuronal survival through its structure and dynamics, mitochondria also produce certain metabolites that are essential for neuronal function (Cioni et al., 2019; Rossoll and Bassell, 2019; Yoon et al., 2012). Apart from producing ATP, mitochondria regulate redox reactions (Osellame et al., 2012) and mitochondrial function is crucial for synaptic transmission (Guo et al., 2017). As per clinical and preclinical studies, mitochondrial dysfunction occurs in the acute phase of ICH and contributes to ICH-induced secondary brain injury mechanisms such as neuroinflammation and oxidative stress (Shao et al., 2022). Moreover, mitochondrial dysfunction is also involved in white matter damage, axonal degeneration and neuronal death post-ICH (Jiang et al., 2019; Smith et al., 2004). In addition, mitochondrial autophagy or mitophagy is a key modulator of cellular responses to environmental stress, cell survival and brain damage after ICH (Chen et al., 2024). However, the precise molecular mechanisms of mitochondrial damage and the extent to which it regulates brain damage after ICH need to be investigated.

**Fig. 8.**
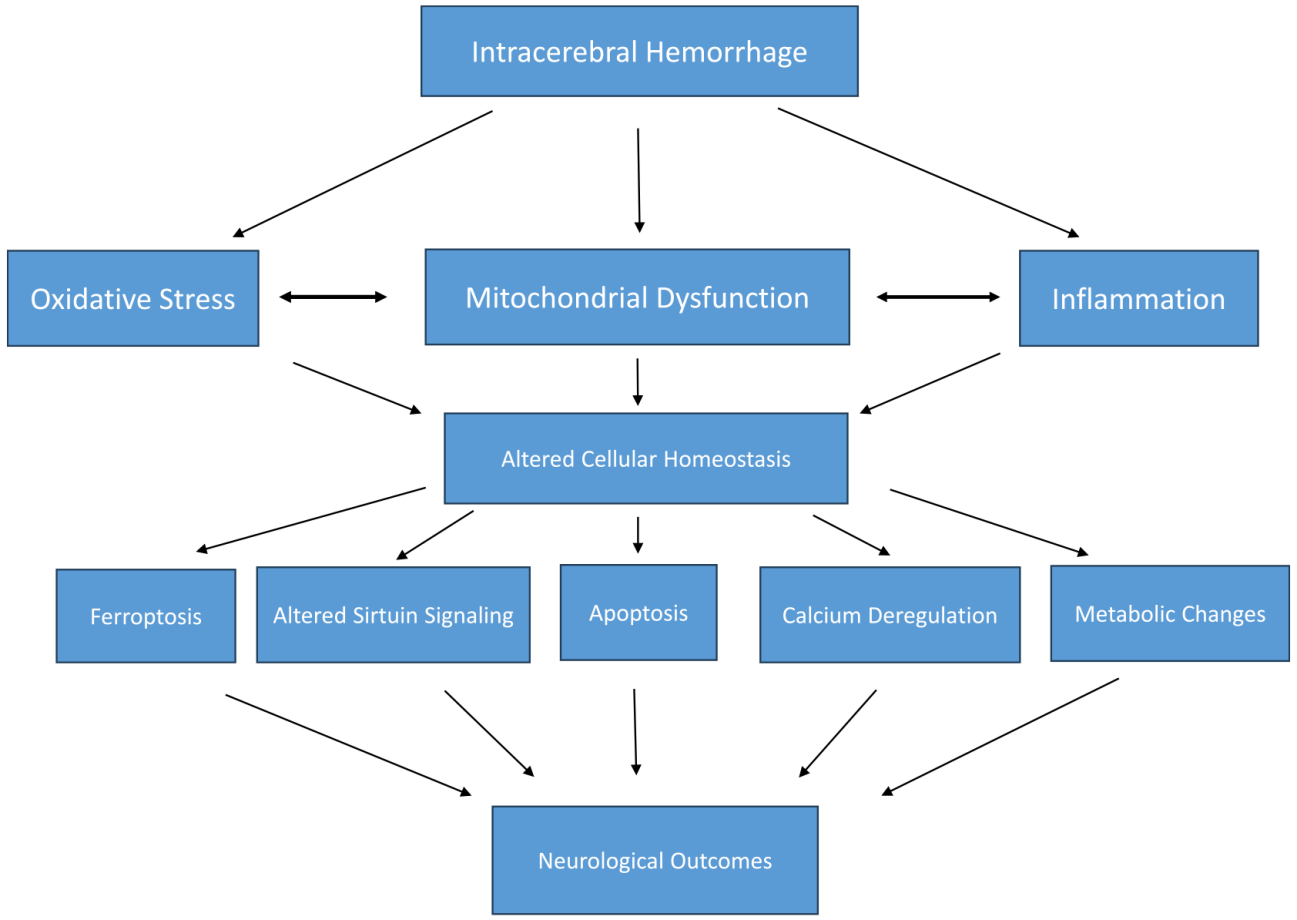
Illustrates the central role of mitochondrial dysfunction in ICH-indued acute brain damage in male and female mice.

Apart from mitochondrial dysfunction, some other prominent pathways associated with the DEPs in both male and female mice brains after ICH per our analysis are the Intrinsic Pathway for Apoptosis, Regulation of TLR by endogenous ligands, 14–3-3-mediated Signaling, Mitochondrial Fatty Acid Beta-Oxidation, Sirtuin Signaling Pathway, Glucose metabolism, and Ferroptosis signaling pathway. Notably, many of these pathological or cellular processes/signaling could emerge from mitochondrial dysfunction, or some could contribute to mitochondrial damage, implicating a central role of mitochondrial dysfunction in ICH-induced brain damage in aged subjects, as depicted in Fig. 8.

Consistent with the bioinformatics analysis, apoptosis is a prominent form of cell death after ICH in the perihematomal region (Qureshi et al., 2003). Hemorrhagic injury-induced changes in mitochondrial homeostasis (Augustynek et al., 2014) can culminate in various types of cell/ neuronal death, including apoptosis (Zhang et al., 2022). In turn, factors released by the dead neurons can activate nearby microglia, the brain’s resident immune cell, to phagocytose the dead neuron or to respond to the brain injury (Jin and Yamashita, 2016). Microglia, upon activation, will secrete many pro-inflammatory cytokines or mediators (Kim and Joh, 2006; Wang et al., 2018), which can further drive apoptosis through direct cellular damage or damage to the mitochondria (Missiroli et al., 2020; Salihu et al., 2016). Furthermore, mitochondrial dysfunction can also lead to a rise in reactive oxygen species levels, causing oxidative stress/damage to the surrounding neural tissue and cell organelles (Mariani et al., 2005; Sidlauskaite et al., 2018; Zheng et al., 2018). Mechanistically, mitochondrial dysfunction is often associated with the abnormal opening of the mitochondrial permeability transition pore (Briston et al., 2017), Ca2+ overload (Friberg and Wieloch, 2002; Norenberg and Rao, 2007), and mitochondrial DNA damage, causing oxidative stress and inflammatory response or NLRP3 inflammasome activation (Shimada et al., 2012; Qiu et al., 2022). Importantly, neuroinflammation (Zhu et al., 2019) and oxidative stress, the critical contributors to ICH-induced cell death and neurological dysfunction (Shao et al., 2022), could also be causatives of mitochondrial dysfunction after ICH and, hence, create a vicious cycle exacerbating brain injury, warranting investigation.

The disruption of mitochondrial function can cause dysregulation of calcium signaling pathways, as mitochondria tightly regulate intracellular calcium compartmentalization, calcium homeostasis, transport, and signaling (Osellame et al., 2012). Mitochondrial calcium levels tend to increase under pathological conditions (Orrenius et al., 2003), disrupting mitochondrial homeostasis (Orrenius et al., 2003). It has been reported that ICH induces perihematomal glutamate receptor activity, increasing intracellular calcium levels (Pivovarova and Andrews, 2010; Wagner, 2007). Furthermore, calcium within the mitochondria can bind to cardiolipin, a phospholipid primarily found in the inner mitochondrial membrane (Orrenius et al., 2003), causing cytochrome *c* release, a key driver of apoptosis (Orrenius et al., 2003). Abnormal calcium signaling has been described as a contributing factor in many neuropathological conditions (Pivovarova and Andrews, 2010; Bezprozvanny, 2009),and its role in the pathophysiology of ICH needs to be evaluated.

Apart from apoptosis, the ferroptosis signaling pathway was identified by the bioinformatics studies. Ferroptosis, characterized by iron-dependent lipid peroxidation (Zille et al., 2017; Li et al., 2023), is an iron-mediated cell death that occurs mostly in the perihematomal region after hemorrhagic stroke. It is often induced by an overload of reactive oxygen species and regulated by mitochondrial iron metabolism, linking ferroptosis closely with mitochondrial dysfunction (Liu et al., 2023a). Also, as per emerging studies, inflammation can cause ferroptosis (Chen et al., 2023), which needs to be explored and investigated after ICH.

Regulation of TLR by endogenous ligands was one of the key pathways identified by bioinformatics analysis. Along these lines, despite the critical role of TLR-4 signaling in ICH-induced neuroinflammation (Fang et al., 2013), the endogenous ligands of TLR-4 after ICH are yet to be fully identified and characterized. To this end, in both male and female aged mice, the expression of S100A9, an endogenous ligand of TLR-4, was found to be significantly upregulated after ICH. S100A9 belongs to a family of calcium-binding proteins with roles in inflammatory signaling pathways (Fritz et al., 2010). Notably, the cerebrospinal fluid level of S100A9 was higher in patients with subarachnoid hemorrhage (SAH) and correlated with the short-term prognosis of patients after SAH (Wang et al., 2024). Also, the neurological outcome was significantly improved in S100A9 knockout mice after SAH (Wang et al., 2024). Apart from its role in inflammation, emerging studies implicate that S100A9 could regulate neurodegeneration, and the pharmacological inhibition of S100A9 attenuated hemin-induced cellular apoptosis (Wang et al., 2024). Also, S100A9 carries amyloidogenic properties and, hence, could contribute to Alzheimer’s Disease pathology (Wang et al., 2014). Further studies are required to identify the functional role of S100A9 after ICH.

Sirtuins belong to the family of class III histone deacetylases (HDACs), which play a role in cell survival, inflammation, and aging (Zhao et al., 2020; Alageel et al., 2018). Sirtuin signaling was also found to be altered after ICH. This finding holds relevance with regard to mitochondrial dysfunction, as SIRT1 signaling has been shown to regulate mitochondrial metabolism and prevent the release of cytochrome *c* after subarachnoid hemorrhage (SAH) (Xu et al., 2021; Zhang et al., 2020). Additionally, SIRT3 has been shown to improve mitochondrial function and reduce neural death after SAH (Wu et al., 2020). However, studies have yet to be conducted to define the role of Sirtuins after ICH.

The functional characterization of DEPs using bioinformatics analysis revealed that the key cellular processes and proteins associated with the DEPs from male and female experimental groups are metabolic processes and enzymes. Also, in line with the role of mitochondria in cellular metabolism, glucose metabolism was one of the prominent pathways identified by the bioinformatics analysis, and aberrant glucose metabolism could result from mitochondrial dysfunction. Notably, altered glucose metabolism has been implicated as a causative of neurodegenerative conditions (Yao et al., 2021),and it can enhance reactive oxygen species levels (Yao et al., 2021), and increase mitochondrial permeability, events that precede apoptosis (Dewanjee et al., 2022). However, despite the clinical study demonstrating the positive correlation between acute plasma glucose levels and poor outcomes after ICH (Tapia-Perez et al., 2014), preclinical evaluations are required to define the precise role of glucose metabolism in the pathophysiology of ICH.

The emerging studies implicate a critical role of lipid metabolism in CNS pathophysiology. However, how ICH alters lipid metabolism and its implications in ICH pathology are largely understudied. The bioinformatics analysis identified LXR/RXR (liver X receptor/retinoid X receptor) activation after ICH, a pathway that can regulate cholesterol efflux. Consistent with this observation, liver X receptor (LXR) agonist enhanced cholesterol efflux and neurological function after ICH in young mice (Zhang et al., 2023). This suggests the potential of therapeutically targeting brain lipid metabolism after ICH, but studies employing old subjects and exploring the underlying molecular mechanisms are highly required. Notably, mitochondria is a key regulator of cellular lipid metabolism. Mitochondrial dysfunction and enhanced levels of reactive oxygen species can cause lipid peroxidation, resulting in cellular damage/dysfunction and contributing to brain pathology (Ayala et al., 2014). Moreover, Mitochondrial Fatty Acid Beta Oxidation is crucial to meet enhanced cellular energy demand upon a brain injury, and its dysfunction can cause neurodegeneration (Labarre et al., 2022). However, its role in ICH pathology remains largely unknown.

Clathrin-mediated endocytosis (CME) and Neutrophil extracellular trap (NET) signaling pathways were found to be modulated after ICH. CME is a major pathway that plays roles in the transport of macromolecules into cells (McMahon and Boucrot, 2011), recycling of synaptic vesicles, and intercellular communication (Mettlen et al., 2018). Also, mitochondrial mitophagy can regulate CME (Zhou et al., 2024). Despite the importance of CME in maintaining cellular homeostasis, the effects of an ICH on CME or vice versa are largely understudied, necessitating investigation. Moreover, endocytosis can trigger neutrophils to release NET (Chen et al., 2012), a fibrous structure of proteins and proinflammatory/oxidized mitochondrial DNA (Brinkmann et al., 2004; Lood et al., 2016). NET is often deployed upon the risk of tissue damage (Papayannopoulos, 2018). Despite this, they have been demonstrated to infiltrate the hematoma after an ICH (Puy et al., 2021), where they can exacerbate the secondary injury due to their proinflammatory effects. As a result, NET has been recognized as a potential therapeutic target for treating ICH (Jin et al., 2022; Liu et al., 2023b). However, studies need to be conducted to elucidate the mechanisms of NET formation after ICH and its regulation of brain damage.

Consistent with the complex pathophysiology of ICH, besides the mechanisms detailed above, the quantitative proteomics analysis revealed significant downregulation of several isoforms of 14–3-3 after ICH in both aged male and female mice in comparison with sham, implicating a possible role of 14–3-3 proteins after ICH. The 14–3-3 proteins are a family of proteins that, in general, interact with proteins that contain specific pSer/pThr motifs (Furukawa et al., 1993; Muslin et al., 1996) and there are seven 14–3-3 isoforms (β, γ, ε, η, ζ, σ, and τ/θ) in mammals (Aitken, 1995; Rosenquist et al., 2000). By interacting with and modulating the target protein functions, 14–3-3 regulates a wide range of cellular processes, including signal transduction, protein trafficking, cell cycle, transcription, and apoptosis (Datta et al., 2000; Fantl et al., 1994; Fu et al., 2000; Peng et al., 1997; Skoulakis and Davis, 1998; Tzivion and Avruch, 2002; Zha et al., 1996). In neurons, 14–3-3 proteins are present in the cytoplasm, intracellular organelles and plasma membrane, and modulate neuronal survival, neuronal signaling, neurite outgrowth, neuronal differentiation, synthesis of neurotransmitters and ion channel regulation (Berg et al., 2003). 14–3-3 proteins regulate neuronal survival in brain pathologies mainly by modulating apoptosis (Zhang et al., 1999). 14–3-3 isoforms can regulate mitochondrial functions (Panda et al., 2024), and their deregulated expression levels are associated with various neuropathological conditions. Notably, some 14–3-3 isoforms interact with proteins such as α-synuclein and β-amyloid that play critical roles in the pathophysiology of Parkinson’s disease (Giusto et al., 2021) and Alzheimer’s disease, respectively (Mateo et al., 2008; McFerrin et al., 2017; Umahara et al., 2004). Moreover, 14–3-3γ, a 14–3-3 isoform, has been implicated in demyelination (Lee et al., 2015) and brain inflammation (Cho and Park, 2020). Importantly, studies have yet to be conducted to define the role of 14–3-3 proteins in the pathophysiology of ICH, particularly in ICH-induced mitochondrial dysfunction, neuroinflammation, and neuronal death.

Besides extravasated blood-induced oxidative brain damage after ICH, aging is often associated with enhanced brain levels of reactive oxygens species (Sohal et al., 1994; Liu et al., 2003; Serrano and Klann, 2004), which can aggravate the disease pathology. While all of the prominent pathways discussed herein were identified in both male and female subjects, as per the proteomics studies, the expression of antioxidant proteins, such as catalase and glutathione peroxidase 1, was upregulated in female-aged mice after ICH but not in males. Moreover, the expression of antioxidant enzyme, superoxide dismutase isoforms, SOD1, and SOD2 was found to be significantly downregulated in response to ICH in aged male mice in comparison with sham. Functionally, SOD1 deletion was associated with enhanced incidence, number and size of brain hemorrhage in a mouse model of spontaneous ICH (Wakisaka et al., 2010), implicating a key role of SOD1 in the pathophysiology of ICH. However, the altered SOD1 and SOD2 expression post-ICH was not observed in the female mice brain, per the proteomics analysis. Consistently, there is a sex-based difference in the expression of *SOD1 and SOD2,* with a higher expression in males (Khymenets et al., 2008). Together, the data suggests sex-based differences in brain oxidative stress responses after ICH, warranting further investigation.

Overall, the present study identified several candidates for functional exploration after ICH and provided comprehensive information on the signaling pathways that are modulated after ICH. Though it would help understand the pathophysiology of ICH that occurs in the aged population, it is not known whether the candidate proteins are exclusive to age-associated pathology and for which further evaluation is required. As per a previous study, healthy age-associated brain proteome changes are predominantly confined to extracellular and synaptic proteins (Tsumagari et al., 2023); however, aging is a complex process affecting several cellular functions, including mitochondrial function. The present study indicates a critical and intertwined role of mitochondrial dysfunction, oxidative stress, and inflammation in the pathophysiology of ICH in both aged male and female subjects. Despite some preclinical studies demonstrating the potential of targeting mitochondria to improve outcomes after ICH, the precise molecular mechanisms that regulate or contribute to mitochondrial dysfunction after ICH, particularly in the context of aging, need to be characterized. Furthermore, sex-based differences in the brain expression of proteins at the basal level (Wingo et al., 2023) could partly be responsible for the observed difference in the DEP profile between male and female subjects after ICH, requiring further studies. However, while most of the key pathways that could play roles in ICH pathology were identified in both male and female subjects, as per the study, there could be sex-specific differences in oxidative stress response to ICH, warranting further investigation. Based on the experimental feasibility and the comprehensive/big data it generates, unbiased approaches like proteomics and bioinformatics could be considered to evaluate the changes in brain protein expression after ICH as part of the functional or therapeutic characterization of protein targets.

## Supplementary Material

Supplementary Material

Supplementary Table 1

Supplementary Table 2

Supplementary Table 3

Supplementary Table 4

Supplementary Table 5

Supplementary Table Legends

Supplementary data to this article can be found online at https://doi.org/10.1016/j.nbd.2025.106936.

## Figures and Tables

**Table 1:** Key signaling pathways and associated DEPs from aged male subjects.

Signaling pathways	Associated proteins	Accession number	Brain injury induced modulation	Fold change
Mitochondrial Dysfunction	ATP synthase subunit g, mitochondrial	Q9CPQ8	↓	0.71
	Aconitate hydratase, mitochondrial	Q99KI0	↓	0.79
	Cytochrome c, somatic	P62897	↓	0.83
	Dihydrolipoyllysine-residue acetyltransferase component of pyruvate dehydrogenase complex, mitochondrial	Q8BMF4	↓	0.69
	Dihydrolipoyl dehydrogenase, mitochondrial	O08749	↓	0.54
	3-hydroxyacyl-CoA dehydrogenase type-2	O08756	↓	0.78
	Isocitrate dehydrogenase [NADP], mitochondrial	P54071	↓	0.50
	Superoxide dismutase 1	P08228	↓	0.62
	Superoxide dismutase mitochondrial	P09671	↓	0.71
Intrinsic Pathway for Apoptosis	Cytochrome c	P62897	↓	0.83
	Calcineurin subunit B type 1	Q63810	↑	1.72
	14–3-3 protein beta/alpha	Q9CQV8	↓	0.61
	14–3-3 protein epsilon	P62259	↓	0.72
	14–3-3 protein gamma	P61982	↓	0.72
	14–3-3 protein eta	P68510	↓	0.56
	14–3-3 protein theta	P68254	↓	0.67
Response to elevated platelet cytosolic Ca2+	Alpha-actinin-1	Q7TPR4	↑	1.52
	Albumin	P07724	↑	7.23
	Endoplasmic reticulum chaperone BiP	P20029	↓	0.80
	Peptidyl-prolyl cis-trans isomerase A	P17742	↓	0.89
	Fibrinogen beta chain	Q8K0E8	↑	42.70
	Fibrinogen alpha chain	E9PV24	↑	10.89
	Fibrinogen gamma chain	Q8VCM7	↑	42.29
	Superoxide dismutase [Cu─Zn]	P08228	↓	0.62
Regulation of TLR by endogenous ligand	Fibrinogen alpha chain	E9PV24	↑	10.89
	Fibrinogen beta chain	Q8K0E8	↑	42.70
	Fibrinogen gamma chain	Q8VCM7	↑	42.29
	Protein S100-A9	P31725	↑	59.24
14–3-3-mediated Signaling	Glial fibrillary acidic protein	P03995	↑	2.22
	14–3-3 protein beta/alpha	Q9CQV8	↓	0.61
	14–3-3 protein epsilon	P62259	↓	0.72
	14–3-3 protein gamma	P61982	↓	0.72
	14–3-3 protein eta	P68510	↓	0.56
	14–3-3 protein theta	P68254	↓	0.67
Neuroinflammation Signaling Pathway	Calbindin	P12658	↓	0.53
	Calretinin	Q08331	↓	0.43
	Glutamate decarboxylase 2	P48320	↓	0.15
	Glutamine synthetase	P15105	↓	0.80
	Calcineurin subunit B type 1	Q63810	↑	1.72
	Superoxide dismutase [Mn]	P09671	↓	0.71
MyD88:MAL(TIRAP) cascade initiated on plasma membrane	Protein S100-A9	P31725	↑	59.24

**Table 2:** Key signaling pathways and associated DEPs from aged female subjects.

Signaling pathways	Associated proteins	Accession number	Brain injury-induced modulation	Fold change
Mitochondrial Dysfunction	Sodium/potassium-transporting ATPase subunit beta-1	P14094	↓	0.73
	ATP synthase subunit gamma, mitochondrial	Q91VR2	↓	0.68
	ATP synthase F(0) complex subunit B1, mitochondrial	Q9CQQ7	↓	0.56
	Calcium/calmodulin-dependent protein kinase type II subunit alpha	P11798	↓	0.77
	Cytochrome c oxidase subunit 4 isoform 1, mitochondrial	P19783	↓	0.58
	Cytochrome c oxidase subunit 6C	Q9CPQ1	↓	0.39
	Cytochrome c, somatic	P62897	↑	1.57
	Dynamin-1-like protein	Q8K1M6	↓	0.51
	Glycerol-3-phosphate dehydrogenase, mitochondrial	Q64521	↓	0.24
	Glutathione peroxidase 1	P11352	↑	11.68
	Maleylacetoacetate isomerase	Q9WVL0	↑	83.65
	NADH dehydrogenase [ubiquinone] 1 alpha subcomplex subunit 10, mitochondrial	Q99LC3	↓	0.61
	NADH dehydrogenase [ubiquinone] 1 alpha subcomplex subunit 11	Q9D8B4	↓	0.31
	NADH dehydrogenase [ubiquinone] 1 alpha subcomplex subunit 5	Q9CPP6	↓	0.30
	NADH dehydrogenase [ubiquinone] 1 alpha subcomplex subunit 9, mitochondrial	Q9DC69	↓	0.40
	NADH dehydrogenase [ubiquinone] 1 beta subcomplex subunit 9	Q9CQJ8	↓	0.15
	NADH-ubiquinone oxidoreductase 75 kDa subunit, mitochondrial	Q91VD9	↓	0.67
	NADH dehydrogenase [ubiquinone] iron-sulfur protein 6, mitochondrial	P52503	↓	0.58
	NADH dehydrogenase [ubiquinone] iron-sulfur protein 7, mitochondrial	Q9DC70	↓	0.30
	NADH dehydrogenase [ubiquinone] iron-sulfur protein 8, mitochondrial	Q8K3J1	↓	0.34
	NADH dehydrogenase [ubiquinone] flavoprotein 1, mitochondrial	Q91YT0	↓	0.53
	NADH dehydrogenase [ubiquinone] flavoprotein 2, mitochondrial	Q9D6J6	↓	0.35
	2-oxoglutarate dehydrogenase, mitochondrial	Q60597	↓	0.69
	Dynamin-like 120 kDa protein, mitochondrial	P58281	↓	0.36
	cAMP-dependent protein kinase catalytic subunit beta	P68181	↓	0.11
	Succinate dehydrogenase [ubiquinone] flavoprotein subunit, mitochondrial	Q8K2B3	↓	0.61
	Cytochrome b-c1 complex subunit 7	Q9D855	↓	0.15
	Cytochrome b-c1 complex subunit 1, mitochondrial	Q9CZ13	↓	0.72
	Cytochrome b-c1 complex subunit 2, mitochondrial	Q9DB77	↓	0.59
	Cytochrome b-c1 complex subunit 8	Q9CQ69	↓	0.61
	Voltage-dependent anion-selective channel protein 1	Q60932	↓	0.56
	Voltage-dependent anion-selective channel protein 2	Q60930	↓	0.55
	Voltage-dependent anion-selective channel protein 3	Q60931	↓	0.48
Sirtuin Signaling Pathway	ATP synthase subunit gamma, mitochondrial	Q91VR2	↓	0.68
	ATP synthase F(0) complex subunit B1, mitochondrial	Q9CQQ7	↓	0.56
	Histone H1.2	P15864	↑	11.34
	Histone H1.4	P43274	↑	4.33
	L-lactate dehydrogenase A chain	P06151	↓	0.81
	NADH dehydrogenase [ubiquinone] 1 alpha subcomplex subunit 10, mitochondrial	Q99LC3	↓	0.61
	NADH dehydrogenase [ubiquinone] 1 alpha subcomplex subunit 11	Q9D8B4	↓	0.31
	NADH dehydrogenase [ubiquinone] 1 alpha subcomplex subunit 5	Q9CPP6	↓	0.30
	NADH dehydrogenase [ubiquinone] 1 alpha subcomplex subunit 9, mitochondrial	Q9DC69	↓	0.40
	NADH dehydrogenase [ubiquinone] 1 beta subcomplex subunit 9	Q9CQJ8	↓	0.15
	NADH-ubiquinone oxidoreductase 75 kDa subunit, mitochondrial	Q91VD9	↓	0.67
	NADH dehydrogenase [ubiquinone] iron-sulfur protein 6, mitochondrial	P52503	↓	0.58
	NADH dehydrogenase [ubiquinone] iron-sulfur protein 7, mitochondrial	Q9DC70	↓	0.30
	NADH dehydrogenase [ubiquinone] iron-sulfur protein 8, mitochondrial	Q8K3J1	↓	0.34
	NADH dehydrogenase [ubiquinone] flavoprotein 1, mitochondrial	Q91YT0	↓	0.53
	NADH dehydrogenase [ubiquinone] flavoprotein 2, mitochondrial	Q9D6J6	↓	0.35
	ATP-dependent 6-phosphofructokinase, muscle type	P47857	↓	0.68
	Succinate dehydrogenase [ubiquinone] flavoprotein subunit, mitochondrial	Q8K2B3	↓	0.61
	ADP/ATP translocase 1	P48962	↓	0.45
	ADP/ATP translocase 2	P51881	↓	0.49
	Solute carrier family 2, facilitated glucose transporter member 1	P17809	↓	0.15
	Tubulin alpha-4A chain	P68368	↓	0.79
	Cytochrome b-c1 complex subunit 2, mitochondrial	Q9DB77	↓	0.59
	Voltage-dependent anion-selective channel protein 1	Q60932	↓	0.56
	Voltage-dependent anion-selective channel protein 2	Q60930	↓	0.55
	Voltage-dependent anion-selective channel protein 3	Q60931	↓	0.48
Neutrophil Extracellular Trap Signaling Pathway	ATP synthase subunit gamma, mitochondrial	Q91VR2	↓	0.68
	ATP synthase F(0) complex subunit B1, mitochondrial	Q9CQQ7	↓	0.56
	Ig gamma-2B chain C region	P01867	↑	107.85
	Immunoglobulin heavy constant mu	P01872	↑	1062.05
	NADH dehydrogenase [ubiquinone] 1 alpha subcomplex subunit 10, mitochondrial	Q99LC3	↓	0.61
	NADH dehydrogenase [ubiquinone] 1 alpha subcomplex subunit 11	Q9D8B4	↓	0.31
	NADH dehydrogenase [ubiquinone] 1 alpha subcomplex subunit 5	Q9CPP6	↓	0.30
	NADH dehydrogenase [ubiquinone] 1 alpha subcomplex subunit 9, mitochondrial	Q9DC69	↓	0.40
	NADH dehydrogenase [ubiquinone] 1 beta subcomplex subunit 9	Q9CQJ8	↓	0.15
	NADH-ubiquinone oxidoreductase 75 kDa subunit, mitochondrial	Q91VD9	↓	0.67
	NADH dehydrogenase [ubiquinone] iron-sulfur protein 6, mitochondrial	P52503	↓	0.58
	NADH dehydrogenase [ubiquinone] iron-sulfur protein 7, mitochondrial	Q9DC70	↓	0.30
	NADH dehydrogenase [ubiquinone] iron-sulfur protein 8, mitochondrial	Q8K3J1	↓	0.34
	NADH dehydrogenase [ubiquinone] flavoprotein 1, mitochondrial	Q91YT0	↓	0.53
	NADH dehydrogenase [ubiquinone] flavoprotein 2, mitochondrial	Q9D6J6	↓	0.35
	Protein disulfide-isomerase A3	P27773	↑	1.38
	Ras-related C3 botulinum toxin substrate 1	P63001	↓	0.59
	Succinate dehydrogenase [ubiquinone] flavoprotein subunit, mitochondrial	Q8K2B3	↓	0.61
	ADP/ATP translocase 1	P48962	↓	0.45
	ADP/ATP translocase 2	P51881	↓	0.49
	Solute carrier family 2, facilitated glucose transporter member 1	P17809	↓	0.15
	Toll-like receptor 7	P58681	↑	83.97
	Cytochrome b-c1 complex subunit 2, mitochondrial	Q9DB77	↓	0.59
	Voltage-dependent anion-selective channel protein 1	Q60932	↓	0.56
	Voltage-dependent anion-selective channel protein 2	Q60930	↓	0.55
	Voltage-dependent anion-selective channel protein 3	Q60931	↓	0.48
Clathrin-mediated Endocytosis Signaling	Albumin	P07724	↑	13.54
	Amphiphysin	Q7TQF7	↓	0.63
	AP-2 complex subunit alpha-1	P17426	↓	0.73
	AP-2 complex subunit alpha-2	P17427	↓	0.56
	AP-2 complex subunit mu	P84091	↓	0.56
	Actin-related protein 2/3 complex subunit 1A	Q9R0Q6	↓	0.55
	Clathrin heavy chain 1	Q68FD5	↓	0.72
	Clusterin	Q06890	↑	1.39
	Dynamin-1	P39053	↓	0.79
	Protein kinase C and casein kinase substrate in neurons protein 1	Q61644	↓	0.72
	Vesicle-associated membrane protein 2	P63044	↓	0.60
	Endophilin-A1	Q62420	↓	0.72
	Synaptojanin-1	Q8CHC4	↓	0.57
	Serotransferrin	Q921I1	↑	11.48
Glucose metabolism	Aspartate aminotransferase, cytoplasmic	P05201	↑	1.14
	Glucose-6-phosphate isomerase	P06745	↓	0.75
	Hexokinase-1	P17710	↓	0.70
	ATP-dependent 6-phosphofructokinase, liver type	P12382	↓	0.62
	ATP-dependent 6-phosphofructokinase, muscle type	P47857	↓	0.68
	Serine/threonine-protein phosphatase 2A 65 kDa regulatory subunit A alpha	Q76MZ3	↓	0.63
	Mitochondrial 2-oxoglutarate/malate carrier protein	Q9CR62	↓	0.46
	Calcium-binding mitochondrial carrier protein Aralar1	Q8BH59	↓	0.55
	Calcium-binding mitochondrial carrier protein Aralar2	Q9QXX4	↓	0.41
Ferroptosis Signaling Pathway	Ferritin light chain 1	P29391	↑	4.09
	Glutaminase liver isoform, mitochondrial	Q571F8	↓	0.29
	Histone H2A type 2-C	Q64523	↑	2.15
	Histone H2A type 2-B	Q64522	↑	1.97
	Histone H2A.Z	P0C0S6	↑	2.47
	Core histone macro-H2A.1	Q9QZQ8	↑	2.50
	Ras-related protein Rap-1A	P62835	↓	0.39
	4F2 cell-surface antigen heavy chain	P10852	↓	0.67
	Serotransferrin	Q921I1	↑	11.48
	Voltage-dependent anion-selective channel protein 2	Q60930	↓	0.55
14–3-3-mediated Signaling	Glial fibrillary acidic protein	P03995	↑	2.04
	Protein disulfide-isomerase A3	P27773	↑	1.38
	Ras-related protein Rap-1A	P62835	↓	0.39
	Tubulin alpha-4A chain	P68368	↓	0.79
	Vimentin	P20152	↑	3.03
	14–3-3 protein beta/alpha	Q9CQV8	↓	0.59
	14–3-3 protein epsilon	P62259	↓	0.71
	14–3-3 protein gamma	P61982	↓	0.68
	14–3-3 protein eta	P68510	↓	0.56
	14–3-3 protein theta	P68254	↓	0.65
	14–3-3 protein zeta/delta	P63101	↓	0.81

## Data Availability

The raw data supporting the study findings are available from the corresponding author upon reasonable request.

## Abbreviations:

AUC: Area under the curve
CME: Clathrin-mediated endocytosis
CNS: Central nervous system
DEPs: Differentially expressed proteins
GFAP: Glial fibrillary acidic protein
GPx1: Glutathione peroxidase 1
ICH: Intracerebral hemorrhage
IHC: Immunohistochemistry
IPA: Ingenuity Pathway Analysis
LC-MS/MS: Liquid Chromatography Tandem-Mass Spectrometry
LXR/RXR: Liver X receptor/retinoid X receptor
NET: Neutrophil extracellular trap
NLRP3: NLR family pyrin domain containing 3
PANTHER: Protein Analysis Through Evolutionary Relationships
PBS: Phosphate buffered saline
PSM: Peptide spectrum match
PVDF: Polyvinylidene difluoride
RIPA: Radio-immunoprecipitation assay
SAH: Subarachnoid hemorrhage
SOD1: Superoxide dismutase [Cu-Zn]
SOD2: Superoxide dismutase [Mn]
TLR: Toll-like receptor
TLR 4: Toll-like receptor 4
