# Supplementary material for: Brain proteome changes after intracerebral hemorrhage in aged male and female mice
Source: Neurobiol Dis. Author manuscript; Available in PMC 2025 Aug 25. (PMC12376891; doi:10.1016/j.nbd.2025.106936)
Supplement: Supplementary Material [file NIHMS2099559-supplement-1.zip › mmc1.pptx]

## Slide 1
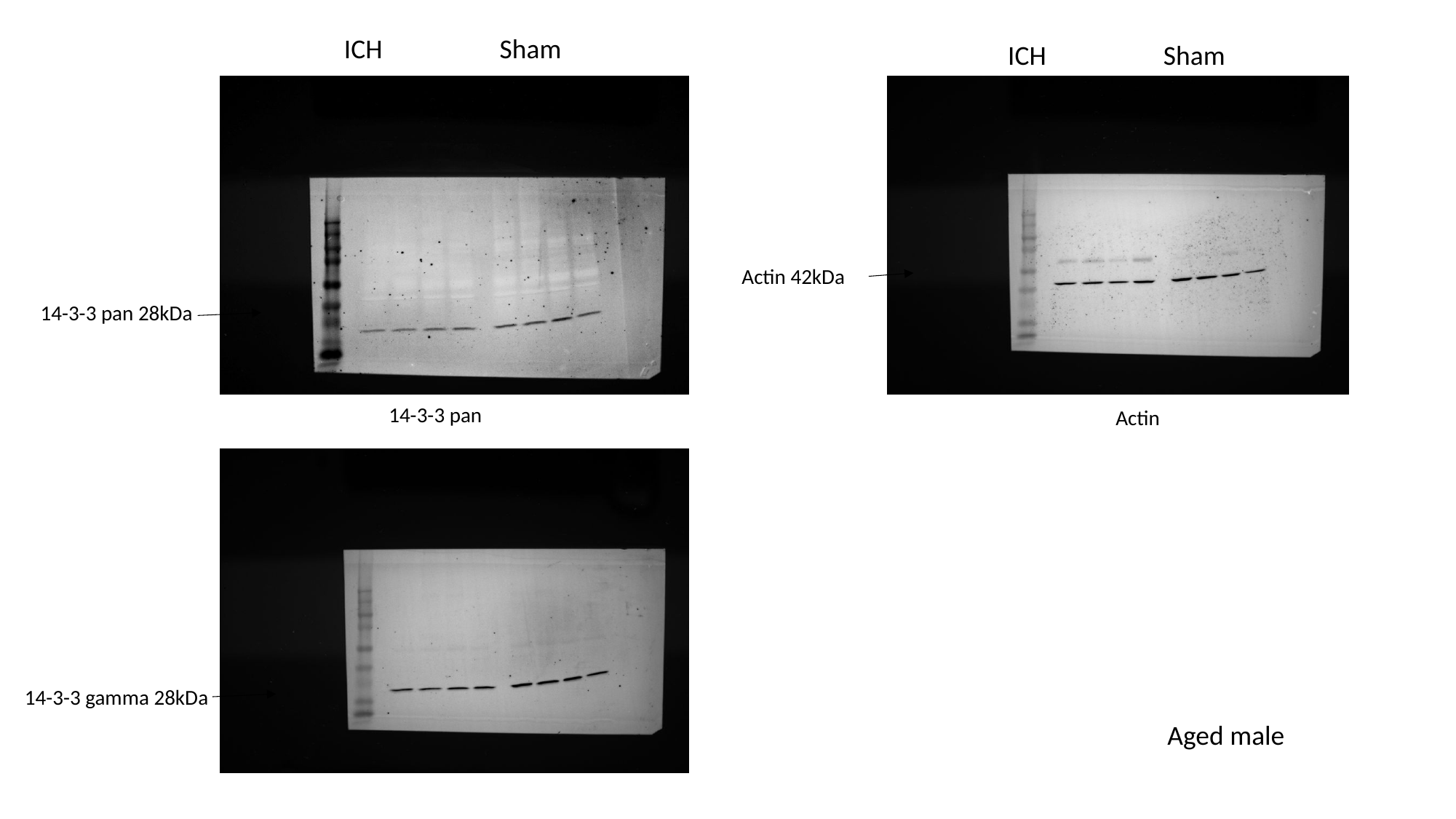

ICH Sham
ICH Sham
Actin 42kDa
14-3-3 pan 28kDa
14-3-3 pan
Actin
14-3-3 gamma 28kDa
Aged male

## Slide 2
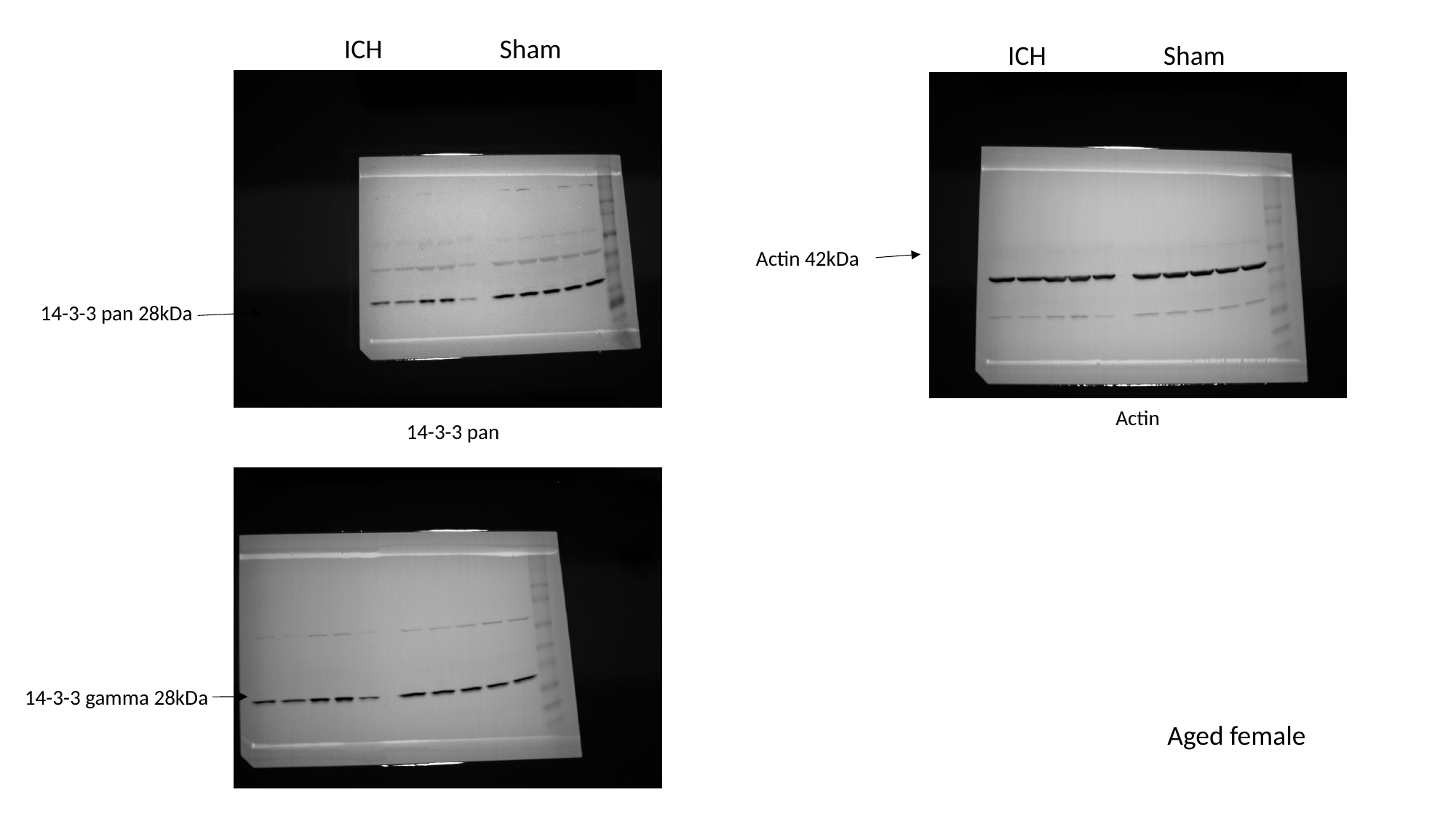

ICH Sham
ICH Sham
Actin 42kDa
14-3-3 pan 28kDa
Actin
14-3-3 pan
14-3-3 gamma 28kDa
Aged female

## Slide 3
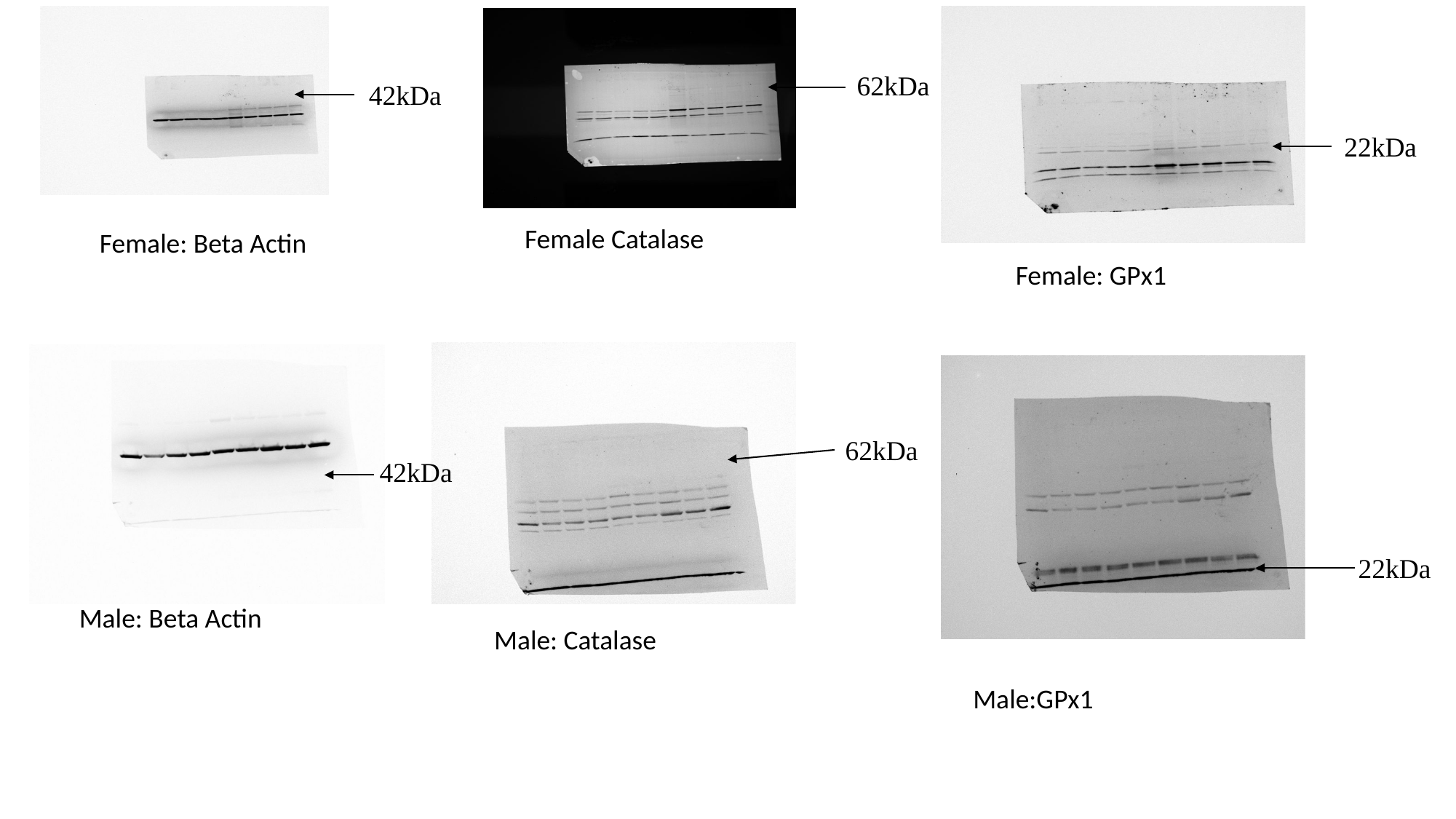

62kDa
42kDa
22kDa
Female Catalase
Female: Beta Actin
Female: GPx1
62kDa
42kDa
22kDa
Male: Beta Actin
Male: Catalase
Male:GPx1
