# Supplementary material for: Brain proteome changes after intracerebral hemorrhage in aged male and female mice
Source: Neurobiol Dis. Author manuscript; Available in PMC 2025 Aug 25. (PMC12376891; doi:10.1016/j.nbd.2025.106936)
Supplement: Supplementary Table Legends [file NIHMS2099559-supplement-7.docx]

Supplementary Table 1: The complete list of DEPs from aged male subjects.

Supplementary Table 2: The complete list of DEPs from aged female subjects.

Supplementary Table 3: The common DEPs from male and female subjects.

Supplementary Table 4: The complete list of canonical pathways associated with the DEPs

from aged male subjects.

Supplementary Table 5: The complete list of canonical pathways associated with the DEPs from aged female subjects.
